# Genetic variant of V825I in the ATP-binding cassette transporter A1 gene and serum lipid levels in the Guangxi Bai Ku Yao and Han populations

**DOI:** 10.1186/1476-511X-10-14

**Published:** 2011-01-19

**Authors:** Xiao-Li Cao, Rui-Xing Yin, Dong-Feng Wu, Lin Miao, Lynn Htet Htet Aung, Xi-Jiang Hu, Qing Li, Ting-Ting Yan, Wei-Xiong Lin, Shang-Ling Pan

**Affiliations:** 1 Department of Cardiology, Institute of Cardiovascular Diseases, the First Affiliated Hospital, Guangxi Medical University, 22 Shuangyong Road, Nanning 530021, Guangxi, People's Republic of China; 2 Department of Neurology, the First Affiliated Hospital, Guangxi Medical University, 22 Shuangyong Road, Nanning 530021, Guangxi, People's Republic of China; 3 Department of Molecular Biology, Medical Scientific Research Center, Guangxi Medical University, 22 Shuangyong Road, Nanning 530021, Guangxi, People's Republic of China; 4 Department of Pathophysiology, School of Premedical Sciences, Guangxi Medical University, Nanning 530021, Guangxi, People's Republic of China

## Abstract

**Background:**

Several genetic variants in the ATP-binding cassette transporter A1 (ABCA1) gene have associated with modifications of serum high-density lipoprotein cholesterol (HDL-C) levels and the susceptibility for coronary heart disease, but the findings are still controversial in diverse racial/ethnic groups. Bai Ku Yao is an isolated subgroup of the Yao minority in southern China. The present study was undertaken to detect the possible association of V825I (rs2066715) polymorphism in the ABCA1 gene and several environmental factors with serum lipid levels in the Guangxi Bai Ku Yao and Han populations.

**Methods:**

A total of 677 subjects of Bai Ku Yao and 646 participants of Han Chinese were randomly selected from our previous stratified randomized cluster samples. Polymerase chain reaction and restriction fragment length polymorphism assay combined with gel electrophoresis were performed for the genotyping of V825I variant, and then confirmed by direct sequencing.

**Results:**

The levels of serum total cholesterol (TC), HDL-C, apolipoprotein (Apo) AI and ApoB were lower in Bai Ku Yao than in Han (*P *< 0.01 for all). The frequency of G and A alleles was 57.4% and 42.6% in Bai Ku Yao, and 57.7% and 42.3% in Han (*P *> 0.05); respectively. The frequency of GG, GA and AA genotypes was 33.7%, 47.4% and 18.9% in Bai Ku Yao, and 33.4%, 48.6% and 18.0% in Han (*P *> 0.05); respectively. There was no difference in the genotypic and allelic frequencies between males and females in the both ethnic groups. The subjects with AA genotype in Bai Ku Yao had higher serum TC levels than the subjects with GG and GA genotypes (*P *< 0.05). The participants with AA genotype in Han had lower serum HDL-C and ApoAI levels than the participants with GG and GA genotypes (*P *< 0.05 for each), but these results were found in males but not in females. Multivariate linear regression analysis showed that the levels of TC in Bai Ku Yao and HDL-C and ApoAI in male Han were correlated with genotypes (*P *< 0.05 for all). Serum lipid parameters were also correlated with sex, age, body mass index, alcohol consumption, and blood pressure in both ethnic groups (*P *< 0.05-0.001).

**Conclusion:**

The present study suggests that the V825I polymorphism in the ABCA1 gene is associated with male serum HDL-C and ApoAI levels in the Han, and serum TC levels in the Bai Ku Yao populations. The difference in the association of V825I polymorphism and serum lipid levels between the two ethnic groups might partly result from different ABCA1 gene-enviromental interactions.

## Introduction

Human and animal model studies have confirmed that abnormal lipid metabolism leads to kinds of diseases, especially altered lipoprotein levels are crucial risk factors for atherosclerosis [1]. A number of studies have established that elevated concentrations of plasma high-density lipoprotein cholesterol (HDL-C) and lower low-density lipoprotein cholesterol (LDL-C) levels reduce the risk for coronary heart disease (CHD) [2-5], but the causality of this inverse association is still incompletely known. There are multiple mechanisms by which HDL-C can be cardioprotective and LDL-C inducing atherogenesis. A widely accepted view is that HDL-C is atheroprotective because of its role in reverse cholesterol transport, a metabolic pathway whereby excess cholesterol in peripheral tissues is transported to the liver for preventing detrimentally accumulation in body [6,7]. It is estimated that up to 60% of the interindividual variation in plasma HDL-C and LDL-C levels is due to genetic variation [8-11], and that the major portion of this variation is polygenic attributable to sequence variation in various loci. It would be interesting to assess the possible association between particular gene variants and serum lipid profiles in the general population.

The ATP-binding cassette transporter A1 (ABCA1) is a member of the large ATP binding cassette transporters family, which comprises proteins translocating a wide variety of substrates across cell membranes utilising ATP [12]. It has been suggested that ABCA1 participates in the efflux of free cholesterol from peripheral cells, including macrophage-derived foam cells, and contributes to the formation of mature HDL by facilitating the lipidation of circulating nascent apolipoprotein (Apo) AI particles with free cholesterol at the plasma membrane [13-15]. Loss-of-function mutations in the ABCA1 gene cause Tangier disease, a rare genetic disorder characterized by near absence of HDL and accumulation of lipids within cells in various tissues including the blood vessel wall [16-19]. ABCA1 mutation carriers have markedly higher incidence of CHD compared with non-carriers [20]. In addition, in families of Tangier disease patients, onset of CHD is significantly earlier in mutation carriers than in noncarriers [21,22]. These results suggest that functional deficiency of ABCA1 could also induce an atherogenic decrease in HDL-C levels, incriminating the ABCA1 gene as a candidate for atherosclerotic complications in the general population [23-25]. A number of single nucleotide polymorphisms (SNPs) have been identified both in the coding and promoter regions of the ABCA1 gene http://www.ncbi.nlm.nih.gov/SNP/. However, for most of them there are no robust data about their functional relevance. A common variant of V825I in the ABCA1 gene is a missense SNP in the exon 17 that locates in the middle part of the protein corresponding to sixth transmembrane α-helix with mutation of GTC→ATC. The ABCA1 V825I polymorphism has been found to be associated with modifications of serum HDL-C levels in some studies [26-29] but not in others [30-33]. Thus, it would be interesting to evaluate whether common genetic variations in the gene constitute a major source of interindividual variability in serum lipid levels and CHD susceptibility in the different ethinc populations.

Han is the largest ethnic group and Yao is the eleventh largest minority among the 55 minority groups in China according to the population size. Bai Ku Yao (White-trouser Yao, all of men wear white knee-length knickerbockers) is an isolated branch of the Yao minority with the population size about 30000. Because of isolation from the other ethnic groups, the special customs and cultures including their clothing, intra-ethnic marriages, dietary habits, and life style are still completely preserved to the present day. In several previous epidemiological studies, we showed that several serum lipid parameters were lower in Bai Ku Yao than in Han Chinese from the same region [34,35]. This ethnic difference in serum lipid profiles remains unknown. We hypothesized that some genetic factors may be different between the two ethnic groups. Therefore, the aim of the present study was to detect the association of V825I polymorphism in the ABCA1 gene and several environmental factors with serum lipid phenotypes in the Guangxi Bai Ku Yao and Han populations.

## Materials and methods

### Study population

A total of 677 participants of Bai Ku Yao and 646 subjects of Han were randomly selected from our previous randomized cluster samples [34,35]. All of them come from both Lihu and Baxu villages in Nandan County, Guangxi Zhuang Autonomous Region, People's Republic of China. They were undergone routine physical examination and appropriate laboratory tests. Ages of the Bai Ku Yao subjects ranged from 15 to 80 years, the average age was 39.74 ± 16.01 years old. There were 324 males (47.86%) and 353 females (52.14%). The mean age of the Han subjects was 41.29 ± 16.39 years (range 15 to 80). There were 315 men (48.76%) and 331 women (51.24%). Both the Han and Bai Ku Yao subjects had no evidence of any chronic illness, including hepatic, renal, or thyroid. The participants with a history of heart attack or myocardial infarction, stroke, congestive heart failure, diabetes or fasting blood glucose ≥ 7.0 mmol/L determined by glucose meter were also excluded. The participants were not taking medications known to affect serum lipid levels (lipid-lowering drugs such as statins or fibrates, beta-blockers, diuretics, or hormones). The present study was approved by the Ethics Committee of the First Affiliated Hospital, Guangxi Medical University. Informed consent was obtained from all participants.

### Epidemiological survey

The epidemiological survey was carried out under internationally standardized methods by following a general protocol [36]. Common information on demographics, socioeconomic status, and lifestyle factors of the participants was collected by answering all the detailed questions of a standardized questionnaire. The data of alcohol consumption included questions about the number of liangs (about 50 g) of rice wine, corn wine, rum, beer, or liquor consumed during the preceding 12 months. Alcohol consumption was categorized into groups of grams of alcohol per day: <25 and ≥ 25. Smoking status was divided into groups of cigarettes per day: <20 and ≥ 20. Several parameters such as height, weight, and waist circumference were measured. Sitting blood pressure was measured three times with the use of a mercury sphygmomanometer after a 5-min rest, and the average of the three measurements was used for the level of blood pressure. Systolic blood pressure was determined by the first Korotkoff sound, and diastolic blood pressure by the fifth Korotkoff sound. Body weight, to the nearest 50 grams, was measured using a portable balance scale. Subjects were weighed without shoes and in a minimum of clothing. Height was measured, to the nearest 0.5 cm, using a portable steel measuring device. From these two measurements body mass index (BMI, kg/m^2^) was calculated.

### Biochemical analyses

Venous blood sample (8 mL) was obtained from each subject between 8 and 11 AM, after 12 hours of fasting. A part of the sample (3 mL) was collected into glass tube and used to determine serum lipid levels. The remaining of the sample (5 mL) was transferred to tubes with anticoagulate solution (4.80 g/L citric acid, 14.70 g/L glucose, and 13.20 g/L tri-sodium citrate) and used to extract DNA. The levels of serum total cholesterol (TC), triglyceride (TG), HDL-C, and LDL-C in samples were detected by enzymatic methods with commercially available kits, Tcho-1, TG-LH (RANDOX Laboratories Ltd., Ardmore, Diamond Road, Crumlin Co. Antrim, United Kingdom, BT29 4QY), Cholestest N HDL, and Cholestest LDL (Daiichi Pure Chemicals Co., Ltd., Tokyo, Japan), respectively. Serum ApoAI and ApoB levels were detected by the immunoturbidimetric immunoassay using a commercial kit (RANDOX Laboratories Ltd.). All determinations were performed with an autoanalyzer (Type 7170A; Hitachi Ltd., Tokyo, Japan) in the Clinical Science Experiment Center of the First Affiliated Hospital, Guangxi Medical University.

### DNA amplification and genetic analysis

Genomic DNA from peripheral blood leukocytes was extracted using the phenol-chloroform method [37-42]. To detect the genetypes, a polymerase chain reaction and restriction fragment length polymorphism (PCR-RFLP) assay was developed [29]. Each 25 μL PCR amplication mixture contained 5-200 ng DNA, 10 × PCR buffer (1.8 mM MgCl_2_) 2.5 μL, 1 U *Taq *polymerase, 2.5 mmol/L of each dNTP (Tiangen, Beijing, People's Republic of China) 2.0 μL, 5 pmol/L of each primer (0.5 μL). The paired primer sequences were: forward, 5'-GGTAGCCCACCACTCCCCTAAAG-3'; reverse, 5'-ATCAGCTGCCTGTCCTTGGACTA-3' (Sangon, Shanghai, People's Republic of China). The PCR amplification reaction was under a cycling protocol of processing started with 95°C for 5 min; and 30 cycles at 94°C for 30 s, 58°C for 45 s and 72°C for 30 s and a final extension at 72°C for 5 min were followed. RFLP assay was performed using 10 U of the appropriate restriction enzyme *Tag*1 per 10 μL PCR products and incubated at 65°C for 1-16 hours as the manufacture described. The genotypes were identified by electrophoresis on 2% agarose gels and visualized with ethidium-bromide staining, ultraviolet illumination. Genotypes were scored by an experienced reader blinded to epidemiological data and serum lipid levels. Six samples (GG, GA and AA genotypes in two; respectively) detected by the PCR-RFLP were also confirmed by sequencing directly. The PCR products were purified by low melting point gel electrophoresis and phenol extraction, and then the DNA sequences were analyzed in Shanghai Sangon Biological Engineering Technology & Services Co., Ltd., People's Republic of China.

### Diagnostic criteria

The normal values of serum TC, TG, HDL-C, LDL-C, ApoAI and ApoB levels, and the ratio of ApoAI to ApoB in our Clinical Science Experiment Center were 3.10-5.17, 0.56-1.70, 0.91-1.81, 2.70-3.20 mmol/L, 1.00-1.78, 0.63-1.14 g/L, and 1.00-2.50; respectively. The individuals with TC >5.17 mmol/L and/or TG >1.70 mmol/L were defined as hyperlipidemic [34,35]. Hypertension was diagnosed according to the criteria of 1999 World Health Organization-International Society of Hypertension Guidelines for the management of hypertension [43,44]. The diagnostic criteria of overweight and obesity were according to the Cooperative Meta-analysis Group of China Obesity Task Force. Normal weight, overweight and obesity were defined as a BMI <24, 24-28, and >28 kg/m^2^; respectively [45].

### Statistical analyses

Epidemiological data were recorded on a pre-designed form and managed with Excel software. All statistical analyses were done with the statistical software package SPSS 17.0 (SPSS Inc., Chicago, Illinois). Quantitative variables were expressed as mean ± standard deviation (serum TG levels were presented as medians and interquartile ranges). Qualitative variables were expressed as percentages. Allele frequency was determined via direct counting, and the standard goodness-of-fit test was used to test the Hardy-Weinberg equilibrium. Difference in genotype distribution between the groups was obtained using the chi-square test. The difference in general characteristics between Bai Ku Yao and Han was tested by the Student's unpaired *t*-test. The association of genotypes and serum lipid parameters was tested by analysis of covariance (ANCOVA). Sex, age, BMI, blood pressure, alcohol consumption, cigarette smoking were adjusted for the statistical analysis. Multivariate linear regression analysis with stepwise modeling was performed to evaluate the association of serum lipid levels with genotypes (GG = 1, GA = 2 and AA = 3) and several environment factors in the combined population of Bai Ku Yao and Han, Bai Ku Yao, Han, males, and females; respectively. A *P *value of less than 0.05 was considered statistically significant.

## Results

### General characteristics and serum lipid levels

The general characteristics and serum lipid levels between the Bai Ku Yao and Han populations are summarised in Table 1. The levels of height, weight, BMI, systolic blood pressure, pulse pressure, serum TC, HDL-C, ApoAI and ApoB were lower in Bai Ku Yao than in Han (*P *< 0.05-0.001), whereas the percentages of subjects who consumed alcohol were higher in Bai Ku Yao than in Han (*P *< 0.001). There were no significant differences in the levels of diastolic blood pressure, serum TG, LDL-C, the ratio of ApoAI to ApoB, age structure, the percentage of subjects who smoked cigarettes, or the ratio of male to female between the two ethnic groups (*P *> 0.05 for all).

**Table 1:** The general characteristics and serum lipid levels between the Bai Ku Yao and Han populations

Parameter	Bai Ku Yao	Han Chinese	***t *(*χ***^**2**^**)**	*P*
Number	677	646	-	-
Male/female	324/353	315/331	0.108	0.783
Age (years)	39.74 ± 16.01	41.29 ± 16.39	-1.734	0.083
Body mass index (kg/m^2^)	22.08 ± 2.39	22.40 ± 3.15	-2.082	0.038
Systolic blood pressure (mmHg)	118.6 ± 17.01	121.70 ± 16.72	-3.294	0.001
Diastolic blood pressure (mmHg)	75.18 ± 9.64	76.27 ± 10.86	-1.936	0.053
Pulse pressure (mmHg)	43.46 ± 12.69	45.44 ± 10.97	-3.026	0.003
Cigarette smoking [n (%)]				
Nonsmoker	477 (70.5)	458 (70.5)		
<20 cigarettes/day	93 (13.0)	84 (13.7)		
≥ 20 cigarettes/day	107 (16.1)	104 (15.8)	0.160	0.923
Alcohol consumption [n (%)]				
Nondrinker	386 (57.0)	406 (62.8)		
<25 g/day	219 (32.3)	105 (16.3)		
≥ 25 g/day	72 (10.6)	135 (20.9)	59.096	0.000
Total cholesterol (mmol/L)	4.31 ± 0.91	4.71 ± 1.01	-7.485	0.000
Triglycerides (mmol/L)	1.00 (0.65)	1.01 (0.64)	-1.1670	0.243
HDL-C (mmol/L)	1.65 ± 0.40	1.88 ± 0.49	-9.271	0.000
LDL-C (mmol/L)	2.55 ± 0.75	2.62 ± 0.76	-1.663	0.097
Apolipoprotein (Apo) AI (g/L)	1.29 ± 0.31	1.41 ± 0.28	-7.067	0.000
ApoB (g/L)	0.84 ± 0.22	0.89 ± 0.23	-4.134	0.000
ApoAI/ApoB	1.65 ± 0.70	1.68 ± 0.57	-4.484	0.628

HDL-C, high-density lipoprotein cholesterol; LDL-C, low-density lipoprotein cholesterol. The value of TG was presented as median (interquartile range). The difference between the two ethnic groups was determined by the Wilcoxon-Mann-Whitney test.

### Electrophoresis and genotypes

**Figure 1:**
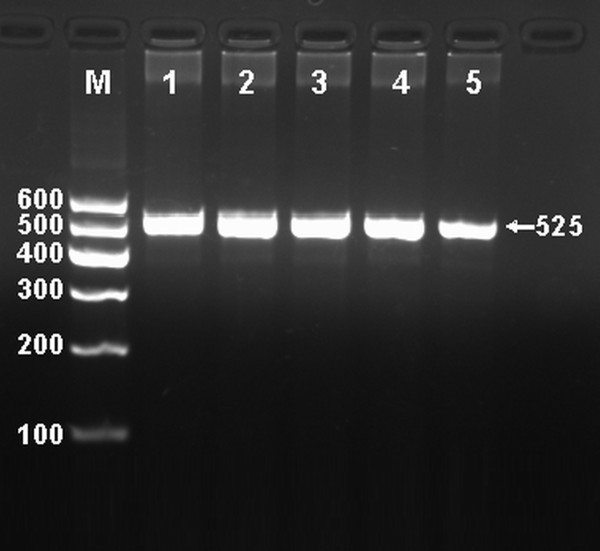
**Electrophoresis of PCR products of the samples**. Lane M, 100 bp marker ladder; lanes 1-5, samples. The 525 bp bands are the target genes.

**Figure 2:**
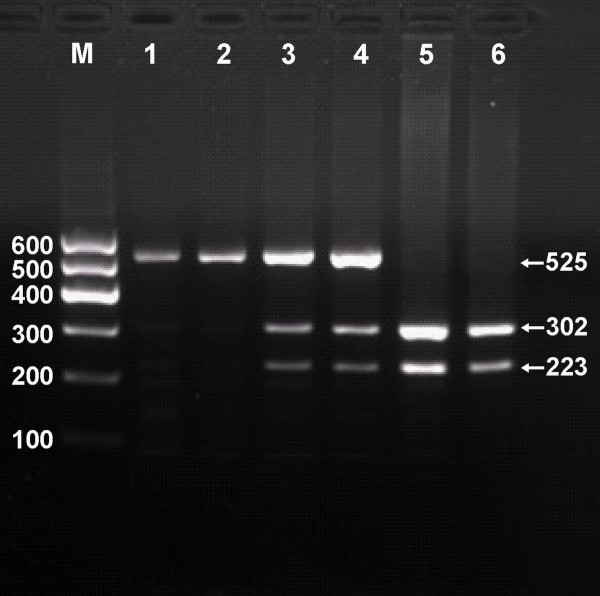
**Genotyping of V825I polymorphism in the ABCA1 gene**. Lane M, 100 bp marker ladder; lanes 1 and 2, GG genotype (525 bp); lanes 3 and 4, GA genotype (525-, 323- and 202-bp); and lanes 5 and 6, AA genotype (323- and 202-bp).

After the genomic DNA of the samples was amplified by PCR and imaged by 2% agarose gel electrophoresis, the purpose gene of 525 bp nucleotide sequences could be found in all samples (Figure 1). The genotypes identified were named according to the presence or absence of the enzyme restriction sites, when a G to A transversion at 825 locus of the ABCA1 gene. The presence of the cutting site indicates the A allele, while its absence indicates the G allele (cannot be cut). Thus, the GG genotype is homozygote for the absence of the site (band at 525 bp), GA genotype is heterozygote for the absence and presence of the site (bands at 525-, 302- and 223-bp), and AA genotype is homozygote for the presence of the site (bands at 302- and 223-bp; Figure 2).

### Genotypic and allelic frequencies

Table 2 gives the genotypic and allelic frequencies of V825I polymorphism in the ABCA1 gene. The frequency of G and A alleles was 57.4% and 42.6% in Bai Ku Yao, and 57.7% and 42.3% in Han (*P *> 0.05); respectively. The frequency of GG, GA and AA genotypes was 33.7%, 47.4% and 18.9% in Bai Ku Yao, and 33.4%, 48.6% and 18.0% in Han (*P *> 0.05); respectively. There was no significant difference in the genotypic and allelic frequencies between males and females in both ethnic groups.

**Table 2:** Genotypic and allelic frequencies of the ABCA1 V825I polymorphism between the Bai Ku Yao and Han populations [n (%)]

Group	n	Genotype			Allele	
		
		GG	GA	AA	G	A
Bai Ku Yao	677	228 (33.7)	321 (47.4)	128 (18.9)	777 (57.4)	577 (42.6)
Han Chinese	646	216 (33.4)	314 (48.6)	116 (18.0)	746 (57.7)	546 (42.3)
*χ*^2^	-		0.265		0.034	
*P*	-		0.876		0.854	
Bai Ku Yao						
Male	324	110 (34.0)	153 (47.2)	61 (18.8)	373 (57.6)	275 (42.4)
Female	353	118 (33.4)	168 (47.6)	67 (19.0)	404 (57.2)	302 (42.8)
*χ*^2^	-		0.210		0.016	
*P*	-		0.990		0.900	
Han Chinese						
Male	315	104 (33.0)	151 (48.9)	57 (18.1)	359 (57.5)	265 (42.5)
Female	331	112 (33.8)	160 (48.3)	59 (17.8)	384 (58.0)	278 (42.0)
*χ*^2^	-	0.490			0.030	
*P*	-	0.976			0.863	

### The nucleotide sequence of V825I polymorphism

**Figure 3:**
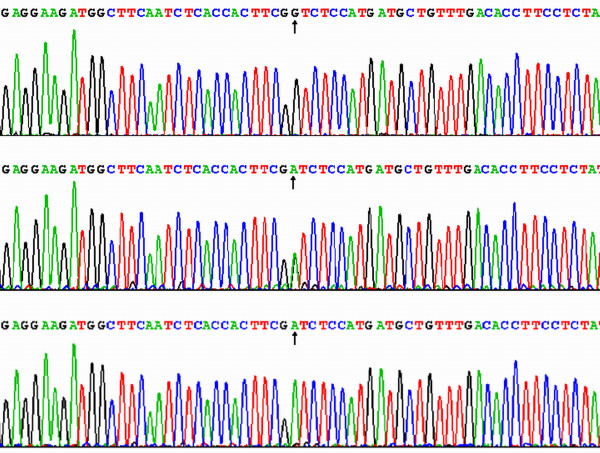
**A part of the nucleotide sequence of the ABCA1 V825I polymorphism**. (A) GG genotype; (B) GA genotype; (C) AA genotype.

The results were shown as GG, GA and AA genotypes by PCR-RFLP, the GG, GA and AA genotypes were also confirmed by sequencing (Figure 3); respectively.

### Genotypes and serum lipid levels

As shown in Table 3, the levels of TC in Bai Ku Yao but not in Han was different among the GG, GA and AA genotypes (*P *< 0.05), the subjects with AA genotype had higher serum TC levels than the subjects with GG and GA genotypes.

**Table 3:** Genotypic frequencies of the ABCA1 V825I polymorphism and serum lipid levels between the Bai Ku Yao and Han populations

Genotype	n	TC (mmol/L)	TG (mmol/L)	HDL-C (mmol/L)	LDL-C (mmol/L)	ApoAI (g/L)	ApoB (g/L)	ApoAI/ApoB
Bai Ku Yao								
GG	228	4.28 ± 0.79	1.09 (0.80)	1.63 ± 0.39	2.52 ± 0.65	1.30 ± 0.34	0.84 ± 0.21	1.65 ± 0.69
GA	321	4.26 ± 0.82	0.96 (0.61)	1.64 ± 0.41	2.52 ± 0.68	1.28 ± 0.29	0.83 ± 0.22	1.64 ± 0.63
AA	128	4.52 ± 1.26	0.95 (0.61)	1.72 ± 0.45	2.69 ± 1.04	1.33 ± 0.35	0.86 ± 0.26	1.73 ± 0.91
*F*	-	3.839	4.621	2.073	2.358	1.460	0.663	0.862
*P*	-	0.022	0.099	0.127	0.095	0.233	0.516	0.423
Male								
GG	110	4.29 ± 0.80	1.25 (0.91)	1.65 ± 045	2.43 ± 0.68	1.36 ± 0.39	0.82 ± 0.21	1.81 ± 0.86
GA	153	4.26 ± 0.88	1.00 (0.66)	1.67 ± 0.45	2.47 ± 0.73	1.32 ± 0.33	081 ± 021	1.76 ± 0.74
AA	61	4.53 ± 1.62	1.02 (0.68)	1.74 ± 0.56	2.63 ± 1.33	1.40 ± 0.44	0.83 ± 0.30	1.96 ± 1.19
*F*	-	1.850	4.969	0.974	1.377	1.040	0.081	1.370
*P*	-	0.159	0.083	0.379	0.254	0.355	0.922	0.256
Female								
GG	118	4.26 ± 0.80	0.97(0.62)	1.60 ± 0.33	2.59 ± 0.62	1.25 ± 0.26	0.87 ± 0.20	1.50 ± 0.44
GA	168	4.26 ± 0.77	0.94(0.56)	1.62 ± 0.36	2.57 ± 0.63	1.24 ± 0.25	0.85 ± 0.22	1.53 ± 0.47
AA	67	4.50 ± 0.80	0.92(0.49)	1.69 ± 0.31	2.73 ± 0.68	1.27 ± 0.22	0.89 ± 0.20	1.51 ± 0.45
*F*	-	2.860	0.595	1.530	2.080	0.474	1.150	0.578
*P*	-	0.059	0.743	0.220	0.126	0.623	0.320	0.560
Han Chinese								
GG	216	4.77 ± 0.99	1.00 (0.57)	1.92 ± 0.51	2.61 ± 0.72	1.43 ± 0.27	0.89 ± 0.22	1.70 ± 0.58
GA	314	4.71 ± 1.06	1.01 (0.68)	1.90 ± 0.48	2.62 ± 0.81	1.42 ± 0.28	0.89 ± 0.24	1.69 ± 0.57
AA	116	4.63 ± 0.90	1.02 (0.70)	1.78 ± 0.48	2.64 ± 0.67	1.36 ± 0.27	0.90 ± 0.21	1.58 ± 0.51
*F*	-	0.600	0.682	3.797	0.190	3.650	0.220	1.930
*P*	-	0.540	0.711	0.023	0.674	0.027	0.800	0.145
Male								
GG	104	4.77 ± 1.03	1.01 (0.59)	1.89 ± 0.55	2.62 ± 0.73	1.42 ± 0.30	0.90 ± 0.23	1.69 ± 0.68
GA	154	4.59 ± 1.13	1.01 (0.64)	1.80 ± 0.49	2.56 ± 0.86	1.36 ± 0.29	0.87 ± 0.25	1.68 ± 0.61
AA	57	4.50 ± 1.04	1.03 (0.78)	1.71 ± 0.49	2.58 ± 0.72	1.32 ± 0.30	0.88 ± 0.22	1.61 ± 0.59
*F*	-	1.039	0.062	3.590	0.037	3.020	0.102	0.575
*P*	-	0.355	0.970	0.029	0.964	0.049	0.903	0.564
Female								
GG	112	4.72 ± 0.96	0.96 (0.56)	1.94 ± 0.47	2.59 ± 0.71	1.43 ± 0.24	0.89 ± 0.21	1.70 ± 0.48
GA	160	4.83 ± 0.97	1.02 (0.77)	1.99 ± 0.45	2.68 ± 0.75	1.43 ± 0.26	0.91 ± 0.22	1.72 ± 0.53
AA	59	4.76 ± 0.75	0.99(0.58)	1.85 ± 0.47	2.70 ± 0.63	1.39 ± 0.23	0.93 ± 0.20	1.55 ± 0.42
*F*	-	0.390	1.182	2.150	0.538	2.640	0.760	2.133
*P*	-	0.677	0.554	0.118	0.585	0.073	0.469	0.120

TC, total cholesterol; TG, triglyceride; HDL-C, high-density lipoprotein cholesterol; LDL-C, low-density lipoprotein cholesterol; ApoAI, apolipoprotein AI; ApoB, apolipoprotein B; ApoAI/ApoB, the ratio of apolipoprotein AI to apolipoprotein B. The value of TG was presented as median (interquartile range). The difference among the genotypes was determined by the Kruskal-Wallis test.

The levels of HDL-C and ApoAI in Han but not in Bai Ku Yao was different among the GG, GA and AA genotypes (*P *< 0.05), the subjects with AA genotype had lower serum HDL-C and ApoAI levels than the subjects with GG and GA genotypes, but these findings were restricted to males but not females.

### Relative factors for serum lipid parameters

Multivariate linear regression analysis showed that the levels of TC in Bai Ku Yao and HDL-C and ApoAI in Han were correlated with genotypes (*P *< 0.05 for all; Table 4). When the multivariate linear regression analysis was performed according to sex in both ethnic groups; respectively, we found that the levels of HDL-C and ApoAI in Han were correlated with genotypes in males but not in females (*P *< 0.05 for each, Table 5). Serum lipid parameters were also correlated with sex, age, BMI, alcohol consumption, cigarette smoking, and blood pressure in both ethnic groups (Tables 4 and 5).

**Table 4:** Correlative factors for serum lipid parameters between the Bai Ku Yao and Han populations

Lipid parameter	Relative factor	Standardized coefficient	Standard error	*t*	*P*
Bai plus Han					
TC	Body mass index	0.100	0.150	2.318	0.021
	Age	0.194	0.002	7.234	0.000
	Ethnic group	-1.159	0.050	-6.200	0.001
	Diastolic blood pressure	0.094	0.003	3.400	0.001
	Weight	0.164	0.006	0.164	0.000
	Sex	0.087	0.061	0.087	0.005
TG	Weight	0.216	0.004	7.889	0.000
	Alcohol consumption	0.114	0.042	4.168	0.000
HDL-C	Age	0.211	0.001	8.214	0.000
	Ethnic group	-0.235	0.023	-9.336	0.000
	Alcohol consumption	0.233	0.018	8.246	0.000
	Sex	0.127	0.026	4.541	0.000
	Body mass index	-0.069	0.004	-2.689	0.007
LDL-C	Body mass index	0.093	0.012	2.097	0.036
	Age	0.188	0.001	6.975	0.000
	Alcohol consumption	-0.090	0.029	-3.092	0.002
	Weight	0.209	0.004	4.412	0.000
	Sex	0.084	0.051	2.491	0.013
ApoAI	Age	0.221	0.001	8.308	0.000
	Alcohol consumption	0.230	0.012	8.126	0.000
	Ethnic group	-0.171	0.015	-6.759	0.000
	Diastolic blood pressure	0.061	0.001	2.272	0.023
	Sex	0.057	0.017	2.021	0.044
ApoB	Body mass index	0.237	0.002	8.922	0.000
	Age	0.160	0.000	5.999	0.000
	Ethnic group	-0.087	0.012	-3.393	0.001
	Diastolic blood pressure	0.105	0.001	3.784	0.000
	Sex	0.081	0.012	3.098	0.002
ApoAI/ApoB	Alcohol consumption	0.165	0.023	6.103	0.000
	Body mass index	-0.155	0.006	-5.722	0.000
Bai Ku Yao					
TC	Body mass index	0.216	0.014	5.821	0.000
	Age	0.139	0.002	3.730	0.000
	Genotype	0.076	0.048	2.051	0.041
TG	Alcohol consumption	0.197	0.068	4.194	0.000
	Body mass index	0.133	0.016	3.505	0.000
	Sex	-0.154	0.099	-3.099	0.002
	Smoking	-0.125	0.066	-2.485	0.013
HDL-C	Alcohol consumption	0.204	0.022	5.502	0.000
	Age	0.177	0.001	4.765	0.000
LDL-C	Body mass index	0.219	0.012	5.812	0.000
	Age	0.114	0.002	3.034	0.003
	Alcohol consumption	-0.080	0.042	-2.107	0.035
ApoAI	Alcohol consumption	0.310	0.017	8.602	0.000
	Age	0.167	0.001	4.639	0.000
ApoB	Alcohol consumption	0.287	0.039	7.748	0.000
	Body mass index	-0.153	0.011	-4.138	0.000
ApoAI/ApoB	Body mass index	0.207	0.004	5.463	0.000
	Age	0.102	0.001	2.674	0.008
	Sex	0.106	0.017	2.800	0.005
	Diastolic blood pressure	0.094	0.001	2.365	0.018
Han Chinese					
TC	Age	0.254	0.002	6.807	0.000
	Diastolic blood pressure	0.128	0.004	3.264	0.001
	Weight	0.281	0.004	7.109	0.000
	Sex	0.165	0.076	4.387	0.000
TG	Weight	0.278	0.005	7.293	0.000
	Alcohol consumption	0.078	0.060	2.033	0.043
HDL-C	Age	0.260	0.001	7.014	0.000
	Alcohol consumption	0.218	0.023	5.628	0.000
	Sex	0.151	0.039	3.770	0.000
	Weight	-0.100	0.002	-2.606	0.009
	Genotype	-0.088	0.025	-2.444	0.015
LDL-C	Age	0.249	0.002	6.620	0.000
	Weight	0.196	0.005	3.524	0.000
	Alcohol consumption	-0.124	0.035	-3.299	0.001
ApoAI	Age	0.307	0.001	8.091	0.000
	Alcohol consumption	0.170	0.013	4.441	0.000
	Sex	0.169	0.021	4.464	0.000
	Diastolic blood pressure	0.085	0.001	2.231	0.026
	Genotype	-0.071	0.014	-1.988	0.047
ApoB	Body mass index	0.261	0.003	6.957	0.000
	Age	0.228	0.001	6.086	0.000
	Diastolic blood pressure	0.106	0.001	2.732	0.006
ApoAI/ApoB	Body mass index	-0.167	0.007	-4.305	0.000

TC, total cholesterol; TG, triglyceride; HDL-C, high-density lipoprotein cholesterol; LDL-C, low-density lipoprotein cholesterol; ApoAI, apolipoprotein AI; ApoB, apolipoprotein B.

**Table 5:** Correlative factors for serum lipid parameters between males and females in both ethnic groups

Lipid parameter	Relative factor	Standardized coefficient	Standard error	*t*	*P*
Bai Ku Yao					
Male					
TC	Body mass index	0.268	0.023	5.036	0.000
	Age	0.130	0.007	2.434	0.015
TG	Body mass index	0.216	0.031	3.973	0.000
HDL-C	Alcohol consumption	0.229	0.037	5.033	0.000
	Age	0.275	0.002	4.286	0.000
	Body mass index	-0.116	0.011	-0.201	0.028
LDL-C	Body mass index	0.261	0.021	4.854	0.000
ApoAI	Alcohol consumption	0.313	0.028	5.887	0.000
	Age	0.187	0.001	3.519	0.000
ApoB	Body mass index	0.293	0.006	5.496	0.000
ApoAI/ApoB	Alcohol consumption	0.272	0.068	4.983	0.000
	Body mass index	-0.188	0.022	-3.449	0.001
Female					
TC	Body mass index	0.170	0.016	3.253	0.001
	Age	0.148	0.003	2.824	0.005
TG	Alcohol consumption	0.205	0.080	3.919	0.000
LDL-C	Body mass index	0.168	0.013	3.209	0.001
	Age	0.162	0.002	3.012	0.002
ApoAI	Age	0.159	0.001	2.300	0.003
	Body mass index	0.160	0.001	2.030	0.022
ApoB	Systolic blood pressure	0.328	0.008	3.067	0.002
	Body mass index	0.202	0.003	3.321	0.001
	Weight	0.025	0.117	2.053	0.041
ApoAI/ApoB	Diastolic blood pressure	-0.155	0.003	-2.937	0.004
Han Chinese					
Male					
TC	Weight	0.334	0.006	6.638	0.000
	Age	0.228	0.003	4.424	0.000
	Alcohol consumption	0.152	0.065	2.932	0.004
TG	Weight	0.385	0.009	7.379	0.000
HDL-C	Age	0.336	0.002	6.729	0.000
	Alcohol consumption	0.309	0.031	6.148	0.000
	Weight	-0.016	0.003	-3.278	0.007
	Genotype	-0.131	0.035	-2.711	0.000
LDL-C	Weight	0.287	0.004	5.389	0.000
	Age	0.174	0.007	3.273	0.001
ApoAI	Age	0.377	0.001	7.609	0.000
	Alcohol consumption	0.278	0.017	5.622	0.000
	Genotype	-0.125	0.020	-2.599	0.010
ApoB	Body mass index	0.295	0.004	5.718	0.000
	Age	0.208	0.001	3.930	0.000
	Smoking	0.124	0.014	2.422	0.016
	Alcohol consumption	0.106	0.015	2.005	0.046
ApoAI/ApoB	Age	0.118	0.020	2.100	0.037
	Body mass index	-0.172	0.011	-3.054	0.002
Female					
TC	Age	0.278	0.007	5.317	0.000
	Weight	0.210	0.006	4.075	0.000
	Diastolic blood pressure	0.152	0.005	2.855	0.005
TG	Diastolic blood pressure	0.167	0.005	3.072	0.002
HDL-C	Age	0.198	0.002	3.658	0.000
LDL-C	Weight	0.317	0.005	6.462	0.000
	Age	0.327	0.002	6.597	0.000
	Alcohol consumption	-0.167	0.052	-3.375	0.001
ApoAI	Age	0.286	0.001	5.433	0.000
	Weight	0.117	0.002	2.220	0.027
ApoB	Body mass index	0.251	0.004	4.878	0.000
	Age	0.240	0.001	4.625	0.000
	Diastolic blood pressure	0.124	0.001	2.344	0.020
ApoAI/ApoB	Weight	-0.188	0.009	-3.463	0.001

TC, total cholesterol; TG, triglyceride; HDL-C, high-density lipoprotein cholesterol; LDL-C, low-density lipoprotein cholesterol; ApoAI, apolipoprotein AI; ApoB, apolipoprotein B.

## Discussion

In the present study, we reported the serum lipid profiles in two Chinese populations, Bai Ku Yao and Han. It offered evidence again to confirm that the levels of serum TC, HDL-C, ApoAI and ApoB were lower in Bai Ku Yao than in Han. The findings were also emphasized in a previous study [34]. There was no significant difference in the levels of serum TG, LDL-C and the ratio of ApoAI to ApoB between the two ethnic groups. It has been confirmed that dyslipidemia is a condition caused by multifactors, including hereditary and modifiabled risk factors. Bai Ku Yao is an isolated subgroup of the Yao minority in China. They reside in two villages, Lihu and Baxu, Nandan County. Both villages are typical infertile mountain area. The main agricultural crops are corn and paddy. Strict intra-ethnic marriages and special diet have been performed in this population from time immemorial. Therefore, the hereditary characteristics and genotypes of some lipid metabolism-related genes in this population may be different from those in Han Chinese.

We showed that the frequency of A allele in Bai Ku Yao (42.6%) was similar to that in Han Chinese (42.3%). There was also no significant difference in the genotypic and allelic frequencies between males and females in the both ethnic groups. However, the frequency of A allele in our study populations was higher than that in a previous report from the Han Chinese [29]. Li *et al*. [29] reported that the frequency of A allele was 32.3% in 386 Han healthy individuals, and the frequency of GG, GA and AA genetypes was 44.9%, 45.5% and 9.6%; respectively. Tan *et al*. [31] showed that the frequency of A allele in male healthy controls in the Singapore Chinese, Malays and Indians was 41.7%, 24.9% and 5.5%; respectively. In a total of 515 Chinese, 112 Malay, 166 Indian male CHD patients, the frequency of A allele was 44.6%, 31.8% and 8.4%; respectively. However, the frequency of A allele in European was very low. It was 6% in Danish general population, 5% in low and 8% in high HDL-C subjects [26,28], 5.6% in participants from Belfast (Northern Ireland) and 6% in those from Glasgow (Scotland) [46], and 8.1% in Dutch [30]. These results indicate that the prevalence of the A allele variation of V825I in the ABCA1 gene may have an ethnic specificity.

The present study also demonstrated that there was a significant association between the A allele and increased serum TC levels in the Bai Ku Yao population and decreased serum HDL-C and ApoAI levels in the male Han population. Conversely, several previous studies found that the V825I polymorphism in the ABCA1 gene was associated with increased serum HDL-C levels [26-29]. Frikke-Schmidt *et al*. [26,28] showed that the A allele carriers had an increased tendency of HDL-C levels in female individuals from the Danish general population but not in males. They thought that the lack of significant association in men for V825I was partly due to less-significant effects on HDL-C in men. Kyriakou *et al*. [27] found that the 825I/825I homozygotes in European ancestry CHD patients had higher mean HDL-C levels than 825V/825I heterozygotes who in turn had higher mean HDL-C levels than 825V/825V homozygotes. The relationships were still observed after adjusting for age, gender, smoking, BMI, hypertension, type 1 diabetes, type 2 diabetes and family history of CHD (*P *= 0.048). In addition, age of symptom onset in 825I/825I homozygotes was 2.71 years higher than that in 825V/825I heterozygotes and 3.76 years higher than that in 825V/825V homozygotes. Li *et al*. [29] found that the V825I polymorphism may affect ApoAI levels in Han Chinese population, but the influence depended on the haplotype generated from V825I and R1587K. The IK carriers had lower serum ApoAI levels than the VR carriers. In the current study, the association of V825I polymorphism and serum ApoAI levels, to some extent at least, was in agreement with a previous study in Han Chinese [29], but the influence on decreased serum HDL-C level in Han Chinese was reverse to that from Danish general population and European ancestry population [26-29]. However, several previous reports failed to find a significant association between the V825I polymorphism in the ABCA1 gene and serum HDL-C levels [30-33]. Clee *et al*. [30] showed that there was no significant difference in the plasma HDL-C levels between the A carriers and noncarriers in CHD patients in a Dutch cohort prospective study, but increased CHD events was observed in the trial (*P *= 0.0008). Tan *et al*. [31] reported that no obviously changes in serum lipid levels were observed in G or A allele carriers in Singapore CHD and CHD-free males (Chinese, Malays and Indian), but the V825I polymorphism clearly associated with CHD status in male Malays. The reason for these conflicting results is not fully understood, probably because of differences in study designs, sample size, race, the methods used to determine serum lipid levels and the polymorphism, as well as gene-enviromental interactions. To our knowledge, the association of the V825I polymorphism and serum TC levels has not been described previously. Thus, further studies are needed to clarify.

Our study also revealed that environmental factors play an important role in serum lipid regulation in the two populations [34]. Multivariate linear regression analysis showed that sex, age, BMI, blood pressure, alcohol consumption, and cigarette smoking were involved in determining serum lipid parameters. The difference in serum lipid profiles between Bai Ku Yao and Han might partly result from different food items and life style factors. The staple food in Bai Ku Yao was corn and the subsidiary foods were rice, soy, buckwheat, sweet potato, and pumpkin products. Approximately 90% of the beverages were corn wine and rum. The alcohol content is about 15% (v/v). They are also accustomed to drink hempseed soup and eat hempseed products. In contrast, the staple food in Han was rice and the subsidiary foods were corn, broomcorn, potato, and taro products. About 90% of the beverage was rice wine. The content of alcohol is about 30% (v/v). A number of experimental and clinical studies have demonstrated that the staple and subsidiary foods and the beverages were more beneficial for serum lipid profiles in Bai Ku Yao than in Han [47-57].

## Conclusion

The present study shows that there is no difference in the genotypic and allelic frequencies between the Han and Bai Ku Yao populations, or between males and females in the both ethnic groups. But the V825I polymorphism in the ABCA1 gene is found to be associated with male serum HDL-C and ApoAI levels in the Han, and serum TC levels in the Bai Ku Yao populations. The participants with AA genotype in Han male had lower serum HDL-C and ApoAI levels than the participants with GG and GA genotypes, whereas the subjects with AA genotype in Bai Ku Yao had higher serum TC levels than the subjects with GG and GA genotypes. The difference in the association of V825I polymorphism and serum lipid levels between the two ethnic groups might partly result from different ABCA1 gene-enviromental interactions.

## Competing interests

The authors declare that they have no competing interests.

## Authors' contributions

XLC participated in the design, undertook genotyping, and drafted the manuscript. RXY conceived the study, participated in the design, carried out the epidemiological survey, collected the samples, and helped to draft the manuscript. DFW, LM, LHHA, XJH, QL and TTY collaborated to the genotyping. WXL and SLP carried out the epidemiological survey, collected the samples, and helped to carry out the genotyping. All authors read and approved the final manuscript.
